# Recurrence Quantification Analysis at work: Quasi-periodicity based interpretation of gait force profiles for patients with Parkinson disease

**DOI:** 10.1038/s41598-018-27369-2

**Published:** 2018-06-14

**Authors:** Ozgur Afsar, Ugur Tirnakli, Norbert Marwan

**Affiliations:** 1 Department of Physics, Faculty of Science, Ege University, 35100 Izmir, Turkey; 2 Centro Brasileiro de Pesquisas Fisicas, Rua Dr. Xavier Sigaud 150, 22290-180 Rio de Janeiro, RJ Brazil; 3 Potsdam Institute for Climate Impact Research, Telegraphenberg A 31, 14473 Potsdam, Germany

## Abstract

In this letter, making use of real gait force profiles of healthy and patient groups with Parkinson disease which have different disease severity in terms of Hoehn-Yahr stage, we calculate various heuristic complexity measures of the recurrence quantification analysis (RQA). Using this technique, we are able to evince that entropy, determinism and average diagonal line length (divergence) measures decrease (increases) with increasing disease severity. We also explain these tendencies using a theoretical model (based on the sine-circle map), so that we clearly relate them to decreasing degree of irrationality of the system as a course of gait’s nature. This enables us to interpret the dynamics of normal/pathological gait and is expected to increase further applications of this technique on gait timings, gait force profiles and combinations of them with various physiological signals.

## Introduction

Parkinson Diesease (PD) is a neuro-degenerative disease which affects gait and mobility related to motor functions. This disease causes functional disorder and death of vital nerve cells producing dopamine being a chemical messenger sending messages to the part of the brain that controls movement and coordination. Decreasing the amount of dopamine in the brain primarily affects mobility of a person and motor control of gait^1^.

The Hoehn-Yahr scale is a commonly used system in order to describe the symptom progression of PD. The scale was originally described in 1967 and included stages 1 through 5^2^. It has been modified with the addition of stages 1.5 and 2.5 to account for the intermediate course of PD in 1990 s^3^. Increasing disease severity of PD increases the score from 0 to 5 where 0 refers to healthy subjects.

In the last years, data sets which include gait variables of patients with various neurological disorders and healthy adults as control group and also comprising their disease stages within the Hoehn-Yahr scale have been widely studied to investigate gait timings or gait force profiles of healthy/patient adults and to establish relations between disease dynamics with them^4–9^. It is of great importance for clinicians^10–13^ to define on the one hand distinct regimes (healthy/patient) and to make comparisons among the degree of order/disorder of the regimes (patients with high/low risk). On the other hand, an optimal classification of disease severity using information theoretic approaches or linear/nonlinear methods^14^ supports the selection of the treatment method, the adjustment of the medication dose, or even the decision about a dopaminergic therapy^15–17^. Although clinicians are widely using subjective/semi-objective measures, such as SF-12 health survey^18^, Short Falls Efficacy Scale-International (Short FES-I)^19^, the visual analog scale (VAS) for pain^20^, UPDRS (parts I, II, and III)^21^, and Hoehn-Yahr staging (the most popular subjective scale used worldwide^22^), objective methods including analyzing methods of gait force profiles/times, postural balance, etc^23^ are available.

The most used objective methods in the literature on identifying gait pathologies and the distinction of PD stages are fractal scaling methods (FSM). In an important research, Bartsch *et al*. reported “a suprisingly result”, with the authors’ own words, using detrended fluctuation analysis (DFA) that is a FSM for the study of long-term fluctuations and correlations in time series^24^. They found the fluctuations in the gait timing were significantly larger for PD patients and early PD patients, who were not treated yet with medications, compared to healthy controls. At the same time, long term correlations of gait force profiles were relatively weak for treated PD patients and healthy controls, while they were significantly larger for early PD patients. Hausdorff and co-workers (a group analyzing gait variabilities such as gait reaction force (GRF), stride time variability (STV), swing interval, etc.) reported using DFA fluctuation dynamics in STV for healthy adults and patients with PD. They originally showed that the data from healthy subjects generally possess fractal indices of around 0.8–1.0 that imply long-range correlated structures and the data of a patient with PD has a scaling exponent with the value is close to 0.5 that is a sign of uncorrelated occurrences^5,25–28^. Kirchner *et al*. proposed an improved fractal analysis^29^, adaptive fractal analysis (AFA), to measure structural information in gait data since AFA is not restricted on the signal being stationary and is a more robust method concerning short time series compared to DFA^30,31^. The second class of methods includes information theoretic approaches. In our previous work, we have shown that the success rate of separation of disease severity is highly related to the Hoehn-Yahr stage of PD patients by applying different spectral-entropy based complexity measures (Shannon, Kullback-Leibler and renormalized entropies)^32^ on data including gait timings and gait force profiles collected by Hausdorff’s group^33^, and, recently, Bernard-Elazari *et al*. discriminated PD patients from healthy older adults (accuracy = 92.3%), mild from severe PD patients (accuracy = 89.8%) and mild PD patients from healthy older adults (accuracy = 85.9%) by applying a machine learning algorithm on the data from PD patients using a body-fixed sensor^34^. A fundamental problem with FSM and spectral-based entropy measures is that they are extremely sensitive to presence of low frequency trends in the data^32,35^. In fact it is easy to replicate the findings of FSM by adding noise to a low frequency sinusoid. Moreover, FSM require long stride-to-stride data to obtain valid results and machine learning is powerful to make predictions or calculated suggestions based on large amounts of data. However, in clinical studies, it is not usual and easy to measure a large number of strides for a patients with PD^29^. In this study, we would like to use a powerful tool, recurrence quantification analysis, for short time series in clinical conditions, and make an objective classification between PD groups using GRF healthy adults and patients with PD as our first aim.

As already shown by Poincaré in 1890^36^, recurrence is one of the fundamental features of dynamical systems and can be used to characterize the specific behaviour of a system in phase space. To statistically expose the characteristic recurrence properties of complex systems, recurrence plots (RPs) have been introduced as a versatile and powerful technique^37^. A RP is a binary matrix whose elements indicate a pair of time points in the phase space those corresponding states are close, i.e., their spatial distance falls below a certain threshold. A quantitative analysis of recurrence plots, namely recurrence quantification analysis (RQA), is very successful to detect distinct regimes and transitions, e.g., regime changes in time series of heart beat intervals^38^, epileptic seizure states^39,40^, climate systems^41^, synthetical systems^42^ etc., in the dynamics of systems from time series by using RP-based heuristic measures. Simplicity and also applicability to short time series makes the technique highly acceptable^43^ in various disciplines such as physics^44,45^, chemistry^46,47^, earth science^48,49^, economy^50,51^ and engineering^52,53^.

It is necessary to define dynamical properties of gait related to both normal and pathological physiological functioning in order to be able to find out distinct phases under gait dynamics. In the literature, extremely regular dynamics is often associated with disease, including periodic (Cheyne-Stokes) breathing, certain abnormally rapid heart rhythms, cyclical blood diseases, epilepsy, neurological tics and tremors^54^. Moreover, the researches on analysis of human gait by using video-based methods on gait sequence^55^, Doppler spectrogram obtained from radar signals revealed from the moving human targets^56^ and phase registration techniques which detected gait periods^57,58^ show that real walking of a human is quasi-periodic. It is also known that some neuro-degenerative disorders which may be caused by some “dynamical diseases” in patients with PD, Huntington’s disease and Tourette’s syndrome show deficits in movement coordination^59^ and may take away the sequence of human gait from normal walking (not pathological) which is quasi-periodic. “Dynamical disease” that leads to abnormal rhythms, which could be either more irregular or more regular than normal, emerge due to alterations in physiological control systems that lead to new stabilities in the dynamics^60^. In the light of all above, we would like to mimic quasi-periodic gait with a theoretical model, sine circle map, and to explain tendencies in complexity measures from RQA from healthy control groups to pathological ones as our second aim.

In this letter, we use real gait force profiles of healthy and patient groups with PD which have different disease severity in terms of Hoehn-Yahr stage to calculate the various heuristic complexity measures coming from RQA. In contrast to linear measures, RQA measures provide deeper insights into more subtle changes of the dynamics. Applying power spectra analysis, variance or mean calculations, the differences in the time series derived from the different disease severities are not as clear (power spectra and variance) or not even significant (see appendix). Using RQA, we are able to evince that entropy, determinism and average diagonal line length (divergence) measures decrease (increases) with increasing disease severity. We also explain these tendencies from normal gait through pathological gait with a theoretical model called sine-circle map^61,62^. Using this model system we clearly show that these tendencies can be explained by changing the degree of irrationality of the system as a course of gait’s nature. This enables us to interpret the dynamics of normal/pathological gait and we hope that it will increase further applications of this technique on gait timings, force profiles and combination of them with various physiological signals.

## Materials and Methods

### Database

We analyze the data set collected by the Hausdorff’s group^33^. They have developed a footswitch system including gait variables of patients with PD and healthy controls. The database which contains measures of gait from 93 patients with idiopathic PD (disease severity: 2, 2.5 and 3, mean age: 66.3 years; 63% men), and 73 healthy controls (disease severity: 0, mean age: 66.3 years; 55% men) is available in “PhysioBank Database”^63^.

The GRF database includes vertical ground reaction force records of subjects when they walk normally (no tasking conditions) on a self-selected pace for approximately 120 seconds on the ground level. Underneath each foot there are 8 sensors that measure force (in Newtons) as a function of time. The output of each of these 16 sensors has been digitized and recorded with Δ*t* = 0.01 (sec). These records also include two signals that reflect the sum of the 8 sensor outputs for each foot^9^.

The data in this database is taken regularly in time (equally sampled) so that no interpolation technique is necessary unlike gait timings (STV, swing time, etc) databases. The RQA analysis needs exactly this kind of data. Moreover, the GRF database is long enough for the spectral analysis.

### Data pre-processing

The database of the Hausdorff’s group can be classified according to disease severity in terms of the Hoehn-Yahr scale of the subjects (10 patients with scale 3 in Group A, 28 patients with scale 2.5 in Group B, 55 patients with scale 2 in Group C and 73 healthy adults with scale 0 in Group D).

**Figure 1:**
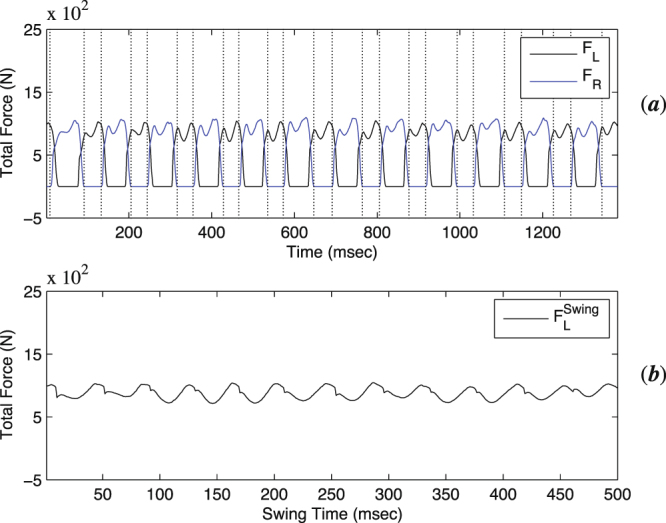
(**a**) Total reaction force under left (*F*_*L*_) and right (*F*_*R*_) foot as the time series. (**b**) Total reaction force under the left foot (${{F}_{L}}^{{\rm{Swing}}}$) whenever the total reaction force under the right foot equals zero during successive swings as a time series.

We use here the data of total reaction force under the left foot ${F}_{L}^{{\rm{Swing}}}$ whenever the total reaction force under the right foot equals zero during successive swings. The corresponding data has length *N* = 500 with total swing time of a foot of 5 sec (Δ*t* = 0.01 (sec)). Swing of a foot means that other foot is in the air and it is strongly correlated with swing of the other foot. Total reaction force during swings presents a quasi-periodic motion for healthy adults whereas it is completely periodic for a sinus oscillation. In Fig. (1a), we plot the time series of total reaction forces under left (*F*_*L*_) and right (*F*_*R*_), whereas in Fig. (1b) we present the same behaviour for total reaction force under the left foot (${F}_{L}^{{\rm{Swing}}}$) whenever the total reaction force under the right foot equals zero during successive swings.

For the RQA, we totally use 40 adults, 10 for each group (A, B, C and D) since the database contains only 10 patients with scale 3. This group of patients with the highest score (scale 3) has also the highest entropy among all groups. Therefore, although we have more than 10 patients in other three stages (stage 0, 2 and 2.5), we also choose 10 patients with highest entropy in these stages so that the chosen ones would have closer entropy values with those of the stage 3 patients. This provides us to see better and to minimize the RQA measure differences in distinct stages.

### Recurrence Quantification Analysis

In a given *m*-dimensional phase space, if the states of two points are sufficiently close to each other, they are considered as recurrent states. Formally, for a given trajectory ${{\bf{x}}}_{i}\,(i=\mathrm{1,}\,\mathrm{2,}\,\mathrm{...,}\,N,\,{\boldsymbol{x}}\in {{\mathbb{R}}}^{m})$ where *N* is the trajectory length, the recurrence matrix is defined by ${R}_{i,j}(\varepsilon )={\rm{\Theta }}(\varepsilon -\Vert {{\boldsymbol{x}}}_{i}-{{\boldsymbol{x}}}_{j}\Vert )$, where *ε* is the neighbourhood threshold, $\Vert \cdot \Vert$ is the Euclidean norm, and Θ(*x*) is the Heaviside step function^37^. If only a one dimensional time series is given, time-delay embedding can be used to reconstruct the phase space trajectory for a time series ${\{{u}_{i}\}}_{i=1}^{N}$^64^, ***x***_*i*_ = (*u*_*i*_, *u*_*i* + τ_, …, *u*_*i* + (*m*−1)τ_), where *m* is the embedding dimension and *τ* is the embedding delay.

We use here the point density based RQA measure, recurrence rate (RR), together with the diagonal structure based RQA measures: entropy (ENT), divergence (DIV), determinism (DET) and average diagonal line length 〈*L*〉^37^. RR is defined by the mean of all elements in the RP

1 $$RR=\frac{1}{{N}^{2}}\sum _{i,j\mathrm{=1}}^{N}{R}_{i,j}\mathrm{.}$$

This is a measure of the density of recurrence points in the RP and corresponds to the correlation sum. The probability to find a diagonal line of exact length *l* in the RP is given by

2 $$p(l)=P(l)/{N}_{l},$$

where *P*(*l*) is histogram of diagonal lines of length *l* and ${N}_{l}={\sum }_{l\ge {l}_{min}}P(l)$ is the total number of diagonal lines. The measure entropy, a measure of the complexity of the RP with regard to the diversity of diagonal lines, is the Shannon entropy of the probability *p*(*l*)

3 $$ENT=-\sum _{l={l}_{min}}^{N}p(l)\mathrm{ln}\,p(l),$$

where *l*_min_ is the minimal diagonal line length (fixed as *l*_min_ = 2 here, because larger *l*_min_ are only necessary for very smooth, continuous data^37^) in the RP. A diagonal line of length *l* means that a segment of the trajectory is rather close during *l* time steps to another segment of the trajectory at a different time; thus these lines are related to the divergence of the trajectory segments. The maximum length of the diagonal structures in the RP, *L*_max_, is defined as *L*_max_ = max({*l*_*i*_; *i* = 1, 2, … *N*_*l*_}), and its inverse

4 $$DIV=\mathrm{1/}{L}_{max}$$

is called divergence (DIV) and related to the largest positive Lyapunov exponent^65^. Processes with uncorrelated or weakly correlated, stochastic or chaotic behaviour cause none or very short diagonals, whereas deterministic processes cause longer diagonals and less single, isolated recurrence points. Therefore, the ratio of recurrence points on the diagonal structures (of at least length *l*_*min*_) to all recurrence points

5 $$DET=\frac{{\sum }_{l={l}_{{\rm{\min }}}}^{N}lP(l)}{{\sum }_{i,\,j}^{N}{R}_{i,j}}\mathrm{.}$$

is a measure for predictability in the system and is called determinism. The average diagonal line length

6 $$\langle L\rangle =\frac{{\sum }_{l={l}_{{\rm{\min }}}}^{N}lP(l)}{{\sum }_{l={l}_{{\rm{\min }}}}^{N}P(l)}\mathrm{.}$$

is the average time that two segments of the trajectory are close to each other, and can be interpreted as the mean prediction time. More details of RQA can be found in Marwan *et al*.^37^.

A parameter specific to the RP is the threshold *ε*. Several criteria for the choice of the threshold value have been advocated in the literature^37^. One approach uses a different threshold *ε*_*i*_ for each state ${\overrightarrow{x}}_{i}$ separately, ensuring a fixed number of neighbours, *N*_*n*_, for every point of the trajectory. This approach is called fixed amount of nearest neighbours (FAN) and corresponds to a RP with the same number of recurrence points in each column and, thus, can be used to preselect the recurrence rate as *RR* = *N*_*n*_/*N*. For RQA of quasiperiodically systems, it has been shown that a recurrence point density in a RP of *RR* = 0.05 would be optimal^66^. Therefore, in this research, all recurrence measures are computed for all data sets (the number of data *N* = 500) with a fixed number of neighbours *N*_*n*_ = 25, ensuring a recurrence rate *RR* of 0.05.

**Figure 2:**
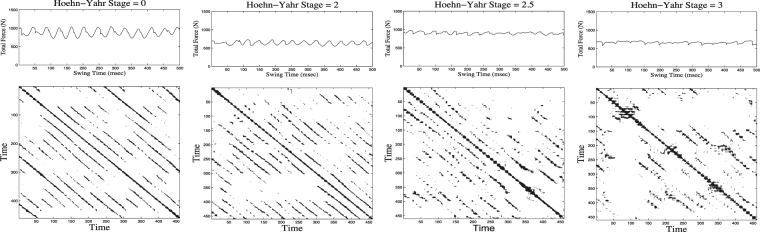
Swing forces (upper) and corresponding recurrence plots (lower) for any adults in every group with disease stages 0, 2, 2.5, 3 from left to the right, respectively.

In Fig. (2) as an example, we plot swing forces (upper panel) and corresponding RPs (lower panel) for selected adults in every group with disease stages 0, 2, 2.5, 3 from left to the right, respectively. For the healthy subject and the subject of low disease stage, the RP consists of a regular, (quasi-)periodic pattern, indicating the more or less periodic gait. With increasing disease stage the patterns in the RP become more disturbed.

The first step in RQA is to reconstruct the *m* dimensional phase space trajectory. The delay time *τ* and the embedding dimension *m* for the analysis are determined using the mutual information and the false nearest neighbours methods. In order to determine the appropriate time delay to be used, the mutual information as a function of time delay *τ* is calculated for each total reaction force data during swings. The optimal values of *τ*, based on the detection of the first local minimum of the mutual information function, vary around 9.99 ± 2.89 (mean and standard deviation) for different data sets. Therefore, the optimal delay time, *τ* = 10 sec, is selected for the phase space reconstruction of the data sets. For the determination of the proper embedding dimension, the percentage of false nearest neighbours for replaced values of the embedding dimension is calculated on each total reaction force data. The optimal embedding dimension *m*, based on the criteria of the percentage of false nearest neighbours being less than 1%, ranges in 4.99 ± 1.40 (mean and standard deviation) for different data sets. Therefore, we choose *m* = 5 for the topologically proper reconstruction of the phase space.

### Statistical analysis

The complexity measures from RQA are subject to the Analysis of Variance (ANOVA) test. For each RQA measure, we use this test to compute the variation between features within a group and between groups with distinct disease severity. When the variation between groups is higher compared to the variation within the group, the feature is considered to be statistically significant (with a significance level of *α* = 0.01 in this work), and, therefore, the *p*-value is very low. As a technical detail for the ANOVA test, we checked whether the values of the RQA measures are homogenous and normally distributed by using the Levene’s test. The test statistic of this test exceeded the significance level (*p*-values of 0.33, 0.09, 0.15 and 0.04 for *ENT*, *DIV*, *DET* and 〈*L*〉, respectively), indicating that the test does not reject the hypothesis and that the RQA measures are normally distributed. We also calculate sensitivity and specificity using the bar plots of all RQA measures with 99% confidence interval in order to check the diagnostic accuracy for the discrimination of patients with PD coming from distinct groups, and also for patients with PD and healthy adults.

### Numerical Example: Sine-circle map

**Figure 3:**
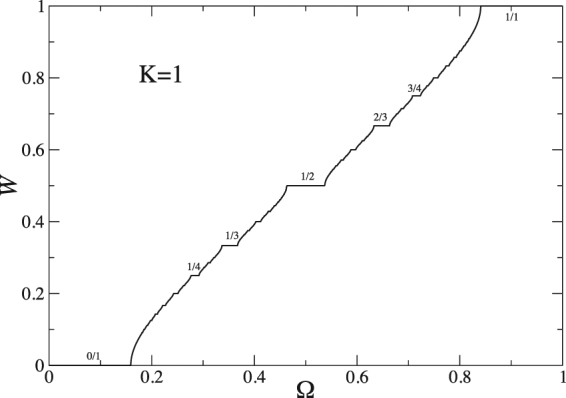
Devil’s staircase which shows the mode-locking structure of the sine-circle map. Some representative rational numbers for which the winding number is locked are given on the curve.

In order to check our results regarding real data, using the sine-circle map^61,62^, we generate quasi-periodic time series to mimic normal walking. This map is well-known in the theory of dynamical systems and defined as

7 $${\theta }_{t+1}={\theta }_{t}+{\rm{\Omega }}-\frac{K}{2\pi }\,\sin \,\mathrm{(2}\pi {\theta }_{t})\,{\rm{m}}{\rm{o}}{\rm{d}}\,\mathrm{(1)},$$

where 0 ≤ *θ*_*t*_ < 1 is a point on a circle and the parameter *K* (with *K* > 0) is a measure of the strength of the nonlinearity. The winding number for this map is defined to be the limit of the ratio

8 $$W=\mathop{\mathrm{lim}}\limits_{t\to \infty }\frac{({\theta }_{t}-{\theta }_{0})}{t},$$

where (*θ*_*t*_ − *θ*_0_) is the angular distance traveled after *t* iterations of the map function. The map develops a cubic inflexion point at *θ* = 0 for *K* = 1. If Ω does not belong to a constant interval (if it is irrational), then *W* is an irrational number and the behavior of the system is quasi-periodic. The winding number *W*(Ω) can be numerically computed from Eq. (8) forming a structure known as the “Devil’s Staircase” shown in Fig. (3). At *K* = 1, where system is at “onset of chaos”, a quasi-periodic time series is generated at special irrational dressed winding numbers which can be approximated by a sequence of truncated continued fractions. The most interesting and well-studied case is the sequence of rational approximants to ${W}_{GM}=(\sqrt{5}-1)/2$ which is called the golden mean and this has the form of an infinite continued-fraction

9 $$W=\frac{1}{1+\frac{1}{1+\frac{1}{1+\mathrm{...}}}}.$$

If the number of fraction lines end at the *n*^th^ denominator, it means the *n* th order approximation to the golden mean. The ratio of Fibonacci numbers *W*_*n*_ = (*F*_*n*_/*F*_*n* + 1_), where *F*_*n*_ is *n* th Fibonacci number and *W*_*n*_ is the *n* th convergent for the golden mean, is also frequently used instead of the infinite continued-fraction. This explains that the sequence of rational numbers *W*_*n*_ converges to the irrational number *W*_*GM*_ as *n* → ∞, yielding the frequency-ratio parameter to approach its limiting value Ω_∞_ (=0.606661063469…)^61,62^.

**Figure 4:**
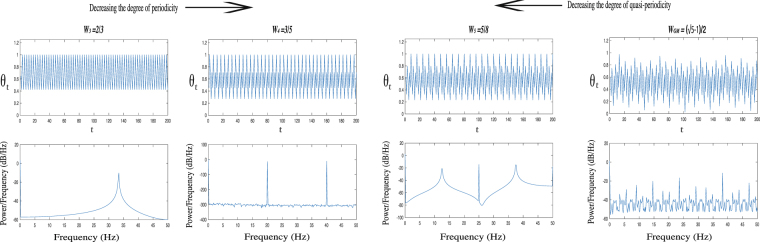
Time series (upper) of the sine-circle map with very low noise density (*D* = 10^14^) and corresponding power spectral densities in four different regimes (for *W*_3_, *W*_4_, *W*_5_, *W*_*GM*_). The degree of irrationality (quasi-periodicity) of the system is increasing from the left one to the right.

Our main aim in this part is to investigate the tendency of the measures of the RQA whenever the system is in quasi-periodic regime corresponding to normal walking and the system is going away from normal walking since it may lead to pathological case. For this purpose, we approach to quasi-periodic regime of the map at *K* = 1 by increasing the degree of the irrationality of the map step by step. To do this, we use the ratios of Fibonacci numbers *W*_*n*_ in order to approach the most irrational number *W*_*GM*_ corresponding to Ω_∞_ as *n* → ∞. Therefore, we iterate the map in four different regimes (for *W*_3_, *W*_4_, *W*_5_, *W*_*GM*_), where the last one is quasi-periodic and the degree of irrationality of the system is increasing from the first one to the last. This case can be shown in Fig. (4) using the time series *θ*_*t*_ (upper) of the map with corresponding power spectral densities (lower) as a frequency (*f*) distribution obtaining from Fourier transformation of time series instead of the residence time distribution. It is also worth noting that number of dominant peaks increase from the left one to the right, that leads to increasing quasi-periodicity. Due to the fact that the real walking is not robotic and it has a noisy nature, we start from *n* = 3 and add white noise with very low density (*D* = 10^14^) to Eq. (7) that does not change the dynamics of the system. This avoids very long plateaues which are extremely regular such as 0/1, 1/1 on the “Devil’s Staircase” shown in Fig. (3), and therefore to better mimic the reality. More precisely, we iterate the map to generate time series with length *N* = 500 after some transient with length 5000 by starting 10 randomly chosen initial conditions and obtain bar plots of the measures from RQA in every specific regimes over these 10 values. This procedure allows us to compare the results with those of the first part which belong to recurrence measures of healthy and patient groups. In the calculation of the measures of the map, all procedures are the same as in the first part for the sake of compatibility except for the choice of embedding dimension and time delay (*m* = 1, *τ* = 1) since it is not necessary for one-dimensional maps^37^.

### Robustness and transition dynamics under noise

In order to check the robustness of the complexity measures from RQA and to show whether there exist transitions between distinct regimes under white noise or not, we increase the noise density added to Eq. (7) from 10^−14^ to 10^−1^ step by step. Then, we calculate median values of the measures and represent them in bar plots for the increasing noise densities and for every winding number from *W*_*GM*_ through *W*_3_ which lead to distinct regimes as irrationality decreases.

## Results

**Figure 5:**
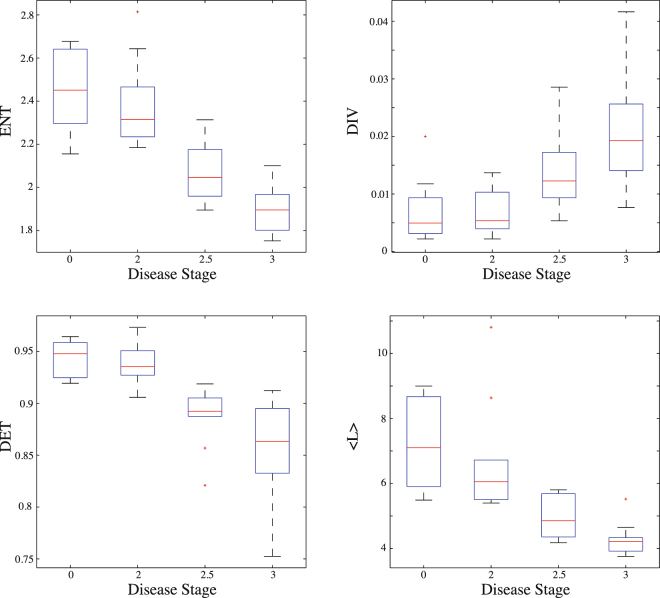
Bar plots of the measures *ENT*, *DIV*, *DET* and 〈*L*〉 calculated from the groups which have disease severity with 0, 2, 2.5, 3, respectively. The lower and upper lines of the box are the 25th and 75th percentiles of the sample, the distance between the top and bottom of the box is the inter quartile range and the line in the middle of the box is the sample median. Outliers (plus sign) are cases with values that are more than 1.5 times the interquartile range.

**Figure 6:**
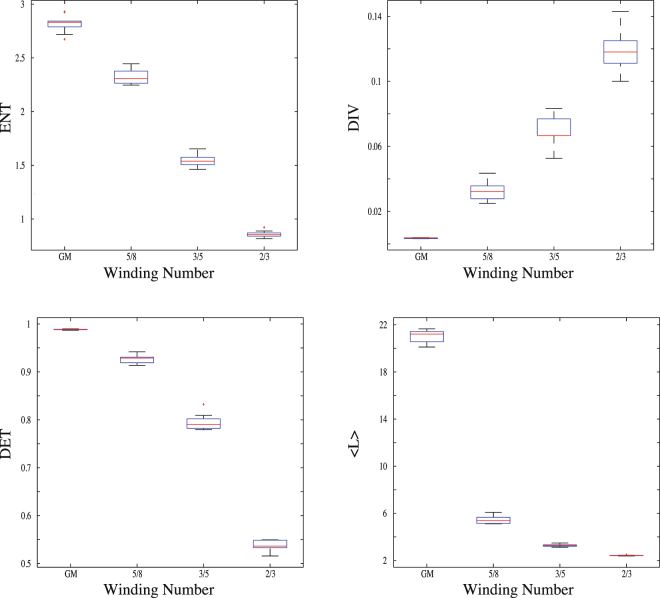
Bar plots of the measures, *ENT*, *DIV*, *DET*, 〈*L*〉, calculated from the groups which belong to different winding numbers *W*_3_, *W*_4_, *W*_5_, *W*_*GM*_, respectively.

In this work, our main aim is to use the above-mentioned measures to evince that three of them (*ENT*, *DET* and 〈*L*〉) decrease whereas the other (*DIV*) increases with increasing disease severity and to explain this tendency using a theoretical model known as the sine-circle map. In order to achieve this task, we calculated the measures *ENT*, *DIV*, *DET* and 〈*L*〉 from the groups which have disease severity with 0, 2, 2.5, 3, respectively (Fig. 5). We find that *DIV* tends to significantly increase with increasing disease stage as *ENT*, *DET* and 〈*L*〉 tend to significantly decrease in terms of their median values. All the median values of RQA measures and the significance levels of groups are summarized in Table 1. An increase of *DIV* and the decrease of *DET* and 〈*L*〉 with changing disease stage from 0 to 3 suggest that this kind of change in disease stage leads to a transition from regular to chaotic state. However, a transition from regular to chaotic regimes cannot result in a decrease of *ENT*, as found in our analysis, since entropy has to increase from ordered state (periodic) to disordered one (chaotic). On the other hand, the decrease in *ENT* is only possible for decreasing degree of irrationality (for increasing degree of periodicity) of a quasi-periodic system that means a transition from quasi-periodic state to periodic one^67^. In order to show this behaviour, we mimic the real data using the sine-circle map in the quasi-periodic regime as shown in Fig. (6), where we show bar plots of the measures, *ENT*, *DIV*, *DET*, 〈*L*〉, calculated from the groups which belong to the different winding numbers *W*_3_, *W*_4_, *W*_5_, *W*_*GM*_, respectively. The degree of irrationality decreases from the left to the right on *x*-axes. It can be shown from the figure that *DIV* tends to increase with decreasing degree of irrationality of the system as *ENT*, *DET* and 〈*L*〉 tend to increase in terms of their median values. These results completely corroborate the results obtained from the real data analysis.

**Table 1:** RQA features (medians and p-values) for distinct disease severities.

*Measures*	*DS* = 0	*DS* = 2	*DS* = 2.5	*DS* = 3	*p*-value
*ENT*	2.4510	2.3149	2.0460	1.8956	<0.0001
*DIV*	0.0049	0.0054	0.0123	0.0193	<0.0001
*DET*	0.9480	0.9356	0.8923	0.8633	<0.0001
〈*L*〉	7.0994	6.0520	4.8556	4.2108	<0.0001

In our statistical test, using all RQA measures in Fig. (5), we obtained a high diagnostic accuracy for discrimination of patients with PD coming from distinct groups. We found a discrimination with a sensitivity of 100% and a specificity of 90% (accuracy = 95%) between the groups with *DS* = 2.5 and *DS* = 2.0, and a sensitivity of 70% and a specificity of 80% (accuracy = 75%) between the groups with *DS* = 3 and *DS* = 2.5. The measures also discriminate the patients with *DS* = 3 and *DS* = 2.5 from healthy adults with a sensitivity of 100% and a specificity of 100% (accuracy = 100%) although the measures can make a discrimination with a sensitivity of 40% and a specificity of 40% (accuracy = 40%) between patients with *DS* = 2 and healthy adults, since the disease stage of this group is very close to the healthy group.

**Figure 7:**
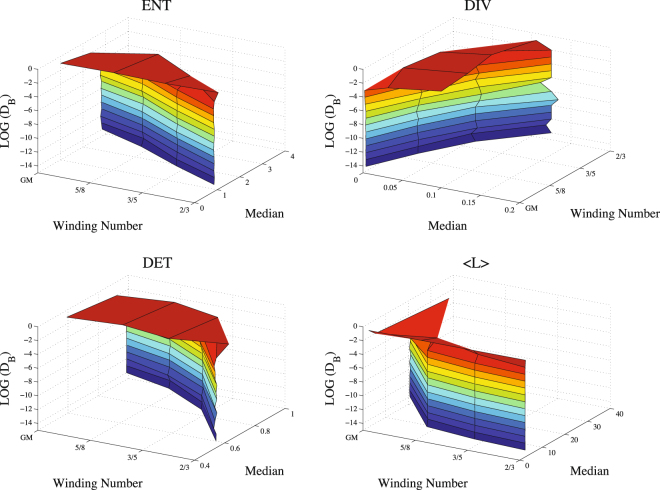
Noise effect to the median values of the measures, *ENT*, *DIV*, *DET*, 〈*L*〉, for decreasing irrationality *W*_*GM*_ through *W*_3_.

Finally, we test the effect of noise, since noise is inevitable for any real system (Fig. 7). This analysis depicts the fluctuations of the median values of the measures, *ENT*, *DIV*, *DET*, 〈*L*〉, for each regime as the noise density is increasing. These results enable us to evaluate the robustness of the measures in terms of their median values after adding noise. We find that all measures are pretty stable in the presence of noise up to very large noise values ($\sim \,{10}^{-4}$). This evidently indicates that all measures used here are very robust under noise.

## Discussion

As disease stage in terms of the Hoehn-Yahr scale increases, complexity measures calculated from RQA using real gait force data present unique tendencies. As *ENT*, *DET* and 〈*L*〉 tend to decrease in terms of their median values, *DIV* tend to increase with increasing disease stage as shown in Fig. (5). The tendency from healthy state with lower divergence, higher entropy and higher determinism values to PD patients with lower values in entropy and determinism and higher values in divergence is a sign that the transitions between disease stages may change from the most irrational state, which is quasi-periodic, to less one. It should be strongly noted that this kind of decreasing degree of irrationality should not be confused with the transition from the chaotic behaviour to periodic one or vice versa. This is just because of the fact that a quasi-periodic trajectory in state space includes both a kind of “order” such as points drifting around the curve which is called a “drift ring” and a “disorder” coming from an incommensurate frequency ratio. Therefore, these trajectories are neither periodic nor chaotic^61,62^. Due to these reasons, the decreasing degree of irrationality in the system may lead to a decrease in the value of entropy and determinism, but an increase in the value of divergence. As a proof of these tendencies, using a quasi-periodic map as a theoretical model and decreasing the degree of irrationality of the system with winding number *W*_*GM*_ through *W*_3_, we show in Fig. (6) that all RQA measures have the same tendency from healthy group to PD patients group within Hoehn-Yahr stage 0 through 3. Therefore, we have shown here that the model system that we have used is totally capable of mimicking the real data in terms of behaviour of the measures coming from RQA and increasing disease stage leads to decreasing of the degree of irrationality of gait.

From a biomechanical and clinical point of view, gait of PD patients looses its automatism and fluidity with a break down of memory of the locomotor control system^68^. From control group to PD patients with increasing pathology, as *DET* and 〈*L*〉 decrease due to the decrease of long-range correlations with disease severity so that the tendencies of these RQA measures are also in accordance with the change of fractal scaling exponents *α* and *H*-values in FSM and AFA^24,29^, the tendency in *ENT* has equivalent outcome with the differentiation of quasi-periodic arrangements and more periodic/less quasi-periodic ones^67^. In accordance with our findings, Pelykh *et al*. in 2015 said that lower entropy values show higher regularity in movement, and higher entropy values show ‘complexity’ and suggested that PD patients’ postural control reflects their rigidity and low adaptability, attesting to their heightened risk for fall injuries^69^. In this paper, we also show that the ‘complexity’ in movement, which leads to higher entropy, results from quasi-periodic fluctuations (not chaotic) in healthy gait. Approaching from quasi-periodic perspective to human movement may also explain why postural sway observed in patients with PD has less complex pattern compared to healthy persons using recurrence quantification analysis (RQA)^70^. Lastly, it is well-known that generally the behaviour of a dynamical system is characterized by the maximum Lyapunov exponent *λ*_max_ which is an index that characterizes the rate of divergence of infinitesimally close trajectories while the system evolves in time and is a kind of order parameter which is inversely proportional of the correlation length^65,71–74^. This completely explains the increasing tendency of the divergence measure *DIV* of RQA from healthy state to more pathological one as opposite of the others which have the decreasing tendencies.

Finally, it is shown in Fig. (7) that the complexity measures which we used in this research are very robust under white noise in the range of noise densities *D*_*B*_ = [10^−14^, 10^−4^]. In this range, the median values of the measures have the same values with very small fluctuations for *W*_*GM*_, *W*_5_, *W*_4_, *W*_3_, which correspond to distinct regimes. The transition between the median values of the regimes emerges only in the range of noise densities *D*_*B*_ = (10^−4^, 10^−1^]. It can be said that noise seems irresistible to the system dynamics in this range which can be called heavy noise.

## Appendix

**Figure 8:**
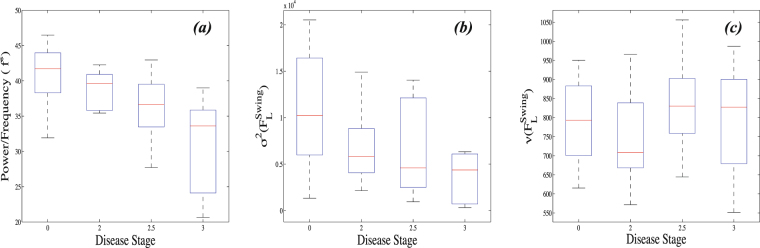
Bar plots of the (**a**) amplitude of the dominant peak in the low frequency region in the range of (0, 5] Hz of power spectral density (*p*-value = 0.002), (**b**) variances (*σ*^2^) (*p*-value = 0.02) and (**c**) means (*μ*) (*p*-value = 0.4) of the swing forces (${F}_{L}^{{\rm{Swing}}}$) calculated from the each adult in distinct groups which have disease severity with 0, 2, 2.5, 3, respectively. We found some differences in the median of the frequency powers and variances between the group with distinct severity 0 through 3. The average values of the swing forces do not show any significant differences. All results are worst than those of RQA in terms of significance level.

We have also applied power spectra, variance and mean analyses (Fig. 8). Slight differences in the frequency spectra (*p*-value = 0.002) are visible between all disease stages, where the difference is rather small between disease stage 0 and 2, but higher between stage 3 and the rest. The differences become less for the variances (*p*-value = 0.02), with almost equal means for the disease stages 2 to 3. Average values of the swing forces are quite similar for all disease stages (*p*-value = 0.4), with slightly elevated values for disease stages 2.5 and 3. Thus, the linear measures are less efficient for discriminating the disease stages. All results in this section are worse than those of RQA measures in terms of significance level (*p*-value < 0.0001 for RQA measures).
